# C-reactive protein and procalcitonin during course of sepsis and septic shock

**DOI:** 10.1007/s11845-023-03385-8

**Published:** 2023-05-19

**Authors:** Tobias Schupp, Kathrin Weidner, Jonas Rusnak, Schanas Jawhar, Jan Forner, Floriana Dulatahu, Jonas Dudda, Lea Marie Brück, Ursula Hoffmann, Thomas Bertsch, Ibrahim Akin, Michael Behnes

**Affiliations:** 1 Department of Cardiology, Angiology, Haemostaseology and Medical Intensive Care, Medical Faculty Mannheim, University Medical Centre Mannheim, Heidelberg University, Heidelberg, Germany; 2 European Center for AngioScience (ECAS), German Center for Cardiovascular Research (DZHK) Partner Site Heidelberg/Mannheim, Mannheim, Germany; 3 Institute of Clinical Chemistry, Laboratory Medicine and Transfusion Medicine, Nuremberg General Hospital, Paracelsus Medical University, Nuremberg, Germany; 4 First Department of Medicine, University Medical Center Mannheim (UMM), Theodor-Kutzer-Ufer 1-3, 68167 Mannheim, Germany

**Keywords:** C-reactive protein, Mortality, Procalcitonin, Prognosis, Sepsis, Septic shock

## Abstract

**Objective:**

The study investigates the diagnostic and prognostic value of C-reactive protein (CRP) and procalcitonin (PCT) in patients with sepsis and septic shock.

**Background:**

Limited data regarding the prognostic value of CRP and PCT during the course of sepsis or septic shock is available.

**Methods:**

Consecutive patients with sepsis and septic shock from 2019 to 2021 were included monocentrically. Blood samples were retrieved from the day of disease onset (day 1), day 2, 3, 5, 7, and 10. Firstly, the diagnostic value of CRP and PCT for the diagnosis of a septic shock, as well as for the discrimination of positive blood cultures, was tested. Secondly, the prognostic value of the CRP and PCT was tested for 30-day all-cause mortality. Statistical analyses included univariable *t*-tests, Spearman’s correlations, C-statistics, and Kaplan–Meier analyses.

**Results:**

A total of 349 patients were included, of which 56% had a sepsis and 44% a septic shock on day 1. The overall rate of all-cause mortality at 30 days was 52%. With an area under the curve (AUC) of 0.861 on day 7 and 0.833 on day 10, the PCT revealed a superior AUC than the CRP (AUC 0.440–0.652) with regard to the discrimination between patients with sepsis and septic shock. In contrast, the prognostic AUCs for 30-day all-cause mortality were poor. Both higher CRP (HR = 0.999; 95% CI 0.998–1.001; *p* = 0.203) and PCT levels (HR = 0.998; 95% CI 0.993–1.003; *p* = 0.500) were not associated with the risk of 30-day all-cause mortality. During the first 10 days of ICU treatment, both CRP and PCT declined irrespective of clinical improvement or impairment.

**Conclusion:**

PCT was a reliable diagnostic tool for the diagnosis of septic shock compared to CRP. Both CRP and PCT were shown to have poor predictive value with regard to 30-day all-cause mortality and were not associated with the risk of all-cause mortality in patients admitted with sepsis or septic shock.

## Introduction

Sepsis represents a major reason for intensive care unit (ICU) admission and is associated with high risk of morbidity and mortality [1]. During the past years, no significant improvement of sepsis-related mortality was observed, although many studies investigated the potential role of novel biomarkers to improve the risk stratification of patients with sepsis and high risk of death [2–5]. Despite the ongoing research to identify biomarkers or combinations of biomarkers with improved accuracy for the diagnosis of sepsis or septic shock, inflammatory markers are not yet included in the diagnosis-making of sepsis within the current sepsis guidelines [6].

C-reactive protein (CRP), discovered in 1930, is a non-specific acute phase protein which may be increased up to 10,000-fold during acute responses to severe infection, sepsis, or major tissue damage [7]. By activating cytotoxic cascades, CRP is involved in the process of removing micro-organisms and necrotic tissue [8]. In clinic routine, CRP is frequently measured as a diagnostic tool for infection, as a marker for disease severity, as well as for the assessment of the therapeutic response (i.e., following antibiotic therapy) [9]. Even in patients with cardiogenic shock, increasing CRP was recently shown to indicate increased short-term mortality [10]. Within a meta-analysis including 9 studies and 1,368 patients, CRP was shown to have a moderate accuracy for the diagnosis of sepsis (area under the curve (AUC) = 0.73), while the diagnostic accuracy of procalcitonin (PCT) was higher (AUC = 0.85) [11]. PCT is a serum peptide with yet unknown function, however, PCT is commonly increased in patients with bacterial infection. Although the PCT was demonstrated to be increased in patients with sepsis within various studies, Tang et al. concluded that the PCT cannot differentiate sepsis from other causes of a systemic inflammatory response syndrome (SIRS) including 18 studies and more than 2,000 patients [12].

Whether CRP and PCT represent good predictors for the severity of a sepsis, as well as the sepsis-related mortality remains controversial [13, 14]. In 2004, our study group suggested no prognostic impact of CRP and PCT measurement in patients with sepsis or septic shock, however, the prognostic role in the current sepsis-3 era needs further investigation [3]. Specifically, the role of the CRP and PCT during the course of a sepsis or septic shock, stratified by disease progression or clinical improvement has not yet been investigated. Therefore, the present study comprehensively investigates the diagnostic and prognostic value of the CRP compared to the PCT in patients admitted to an internistic ICU with sepsis or septic shock.

## Methods

### Study patients, design, and data collection

The present study prospectively included all consecutive patients presenting with sepsis or septic shock on admission to the internistic ICU at the University Medical Center Mannheim, Germany, from June 2019 to January 2021 as recently published [15]. The presence of sepsis and septic shock, as well as important laboratory data, sepsis-related scores, hemodynamic measurements, ventilation parameters were assessed on disease onset (i.e., day 1), as well as on day 2, 3, 5, 7, and 10. Further documented data contained baseline characteristics, prior medical history, length of index hospital stay, data derived from imaging diagnostics, as well as pharmacological therapies. Documentation of source data was performed by intensivists and ICU nurses during routine clinical care.

The present study derived from an analysis of the “Mannheim Registry for Sepsis and Septic Shock” (MARSS-registry), which represents a prospective single-center registry including consecutive patients presenting with sepsis or septic shock, who were acutely admitted to the ICU for internal medicine of the University Medical Center Mannheim (UMM), Germany (clinicaltrials.gov identifier: NCT05231720). The registry was carried out according to the principles of the declaration of Helsinki and was approved by the medical ethics committee II of the Medical Faculty Mannheim, University of Heidelberg, Germany.

### Inclusion and exclusion criteria, study endpoints

For the present study, all consecutive patients with sepsis and septic shock were included. Patients without CRP measurement on day 1 were excluded from the present study. No further exclusion criteria were applied. The diagnosis of sepsis and septic shock was determined according to the “Third International Consensus Definition for Sepsis and Septic Shock” (i.e., sepsis-3) [6]. Accordingly, sepsis was defined as life-threatening organ dysfunction, caused by a dysregulated host response to infection. Organ dysfunction is defined as an increase of ≥ 2 in the Sequential Organ Failure Assessment (SOFA) score. Septic shock was defined as persistent hypotension, despite adequate volume resuscitation, requiring vasopressors to maintain a mean arterial pressure (MAP) ≥ 65 mm Hg and a lactate ≥ 2 mmol/l [6].

All-cause mortality at 30 days was documented using our electronic hospital information system and by directly contacting state resident registration offices (‘bureau of mortality statistics’). Identification of patients was verified by place of name, surname, day of birth, and registered living address. No patient was lost to follow-up with regard all-cause mortality at 30 days.

### Risk stratification

Firstly, the diagnostic and prognostic value of the CRP and PCT was investigated within the entire study cohort. Thereafter, the patients were stratified for initial sepsis and initial septic shock.

To investigate the prognostic role of the CRP and PCT stratified for dynamic disease progression and clinical improvement, the study group was divided into the following groups: (group 1) patients with initial sepsis without progression to septic shock according to current international guidelines [6]; (group 2) patients with initial sepsis with progression to septic shock; (group 3) patients with initial septic shock with clinical improvement to sepsis without shock; (group 4) patients with initial septic shock without clinical improvement. For this sub-analysis, patients were re-classified to sepsis without shock and septic shock according to the sepsis-3 criteria on the analyzed ICU treatment days (i.e., on days 1, 2, 3, 5, 7, and 10).

### Statistical methods

Quantitative data is presented as mean ± standard error of mean (SEM), median, and interquartile range (IQR), and ranges depending on the distribution of the data. They were compared using the Student’s *t*-test for normally distributed data or the Mann–Whitney U test for nonparametric data. Deviations from a Gaussian distribution were tested by the Kolmogorov–Smirnov test. Qualitative data is presented as absolute and relative frequencies and was compared using the Chi-square test or the Fisher’s exact test, as appropriate. Box plots for CRP and PCT were created for the comparisons of patients with sepsis and septic shock during the first week of sepsis on days 1, 3, 5, 7, and 10. Spearman’s rank correlation for nonparametric data was used to test the association of the CRP and PCT with medical and laboratory parameters on day 1.

#### Diagnostic performance of CRP and PCT

C-statistics were applied with calculation of the receiver operating characteristic (ROC) and the corresponding area under the curve (AUC) within the entire cohort to assess the discriminative performance of the CRP and PCT with regard to the diagnosis of septic shock and sepsis at days 1, 3, 5, 7, and 10. Thereafter, the diagnostic accuracy of CRP and PCT with regard to blood-culture confirmed sepsis was tested. AUCs for the diagnostic performance were compared by the method of Hanley and McNeil [16].

#### Prognostic performance of CRP and PCT

C-statistics were applied with calculation of ROC and the corresponding AUC within the entire cohort at days 1, 3, 5, 7, and 10 with regard to the 30-day all-cause mortality. AUCs for prognostic performance were compared by the method of Hanley and McNeil [16].

Kaplan–Meier analyses according to the median CRP and PCT levels were performed within the entire study cohort, as well as separated by clinical improvement or impairment (i.e., groups 1–4). Univariable hazard ratios (HR) were given together with 95% confidence intervals.

Results of all statistical tests were considered significant for p ≤ 0.05. SPSS (Version 25, IBM, Armonk, New York) and GraphPad Prism (Version 9, GraphPad Software, San Diego, California) were used for statistics.

## Results

### Study population

From a total of 361 consecutive patients with sepsis or septic shock, 12 patients without CRP measurement on day 1 were excluded. The final study cohort comprised of 349 patients with sepsis or septic shock on admission. 56% of the patients presented with a sepsis and 44% with a septic shock on day 1. The median CRP level on day 1 was 144 mg/l (IQR 83–220 mg/l). PCT was measured in 270 patients (77%) on day 1 (median PCT level 2.8 ng/ml (IQR 0.7–18.4 ng/ml)). As seen in Table 1, patients were median-aged at 69 years and most patients were males (64%). When stratified for patients presenting with sepsis or septic shock (Table 1, middle and right panel), cardiovascular risk factors (including arterial hypertension, diabetes mellitus, and hyperlipidaemia) did not differ among patients with sepsis or septic shock on admission. Furthermore, the rates of coronary artery disease (31% vs. 36%; *p* = 0.305), heart failure (17% vs. 23%; *p* = 0.142), and atrial fibrillation (27% vs. 29%; *p* = 0.673) were comparable in both groups. Of note, left ventricular ejection fraction (LVEF) < 35% was more frequently observed in patients with septic shock (20% vs. 11%; *p* = 0.001) with increased rates of cardiopulmonary resuscitation (20% vs. 6%; *p* = 0.001).

**Table 1:** Baseline characteristics

	**All patients** (*n* = 349)		**Sepsis** (*n* = 197)		**Septic shock** (*n* = 152)		***p*-value**
**Age**, median; (IQR)	69	(60–79)	69	(60–79)	70	(58–80)	0.622
**Male sex,** *n* (%)	222	(63.6)	128	(65.0)	94	(61.8)	0.546
**Body mass index** (kg/m^2^)**,** median; (IQR)	26.23	(23.44–29.41)	26.3	(22.93–29.39)	26.23	(23.67–30.04)	0.994
**Entry criteria,** median; (IQR)							
Body temperature (°C)	36.7	(36–37.5)	36.9	(36–37.6)	36.4	(35.7–37.3)	**0.014**
Heart rate (bpm)	99	(86–115)	97	(85–112)	101	(87–119)	0.088
Systolic blood pressure (mmHg)	109	(94–130)	112	(99–133)	103	(88–125)	**0.001**
Respiratory rate (breaths/minute)	21	(18–26)	22	(18–26)	20	(17–27)	0.690
**Cardiovascular risk factors,** *n* (%)							
Arterial hypertension	225	(64.5)	128	(65.0)	97	(63.8)	0.823
Diabetes mellitus	119	(34.1)	64	(32.5)	55	(36.2)	0.470
Hyperlipidemia	99	(28.4)	50	(25.4)	49	(32.2)	0.159
Smoking	99	(28.4)	55	(27.9)	44	(28.9)	0.833
**Prior medical history,** *n* (%)							
Coronary artery disease	116	(33.2)	61	(31.0)	55	(36.2)	0.305
Congestive heart failure	68	(19.5)	33	(16.8)	35	(23.0)	0.142
Atrial fibrillation	97	(27.8)	53	(26.9)	44	(28.9)	0.673
Chronic kidney disease	69	(19.8)	43	(21.8)	26	(17.1)	0.272
COPD	64	(18.3)	39	(19.8)	25	(16.4)	0.423
Liver cirrhosis	33	(9.5)	15	(7.6)	18	(11.8)	0.181
Malignancy	113	(32.4)	54	(27.4)	59	(38.8)	**0.024**
Immunosuppression	47	(13.5)	29	(14.7)	18	(11.8)	0.435
**LVEF at admission, *n* (%)**							
≥ 55%	134	(41.6)	81	(44.0)	53	(38.4)	**0.001**
54–45	89	(27.6)	62	(33.7)	27	(19.6)	
44–35%	51	(15.8)	20	(10.9)	31	(22.5)	
< 35%	48	(14.9)	21	(11.4)	27	(19.6)	
Not documented	27	-	13	-	14	-	
**Cardiopulmonary resuscitation**, *n* (%)	41	(11.7)	11	(5.6)	30	(19.7)	**0.001**
In-hospital	12	(3.4)	3	(1.5)	9	(5.9)	**0.001**
Out-of-hospital	29	(8.3)	8	(4.1)	21	(13.8)	

*COPD* chronic obstructive pulmonary disease, *IQR* interquartile range, *LVEF* left ventricular ejection fraction

Level of significance *p* < 0.05. Bold type indicates statistical significance

Sepsis-related data, laboratory data, and sepsis-related outcomes are outlined within Table 2. Established sepsis-scores were higher in patients presenting with septic shock compared to patients presenting with sepsis, including the acute physiology score (median 18 vs. 14; *p* = 0.001), acute physiology and chronic health evaluation II (APACHE II) score (median 26 vs. 22; *p* = 0.001) and SOFA score (median 13 vs. 10; *p* = 0.001). In both groups (i.e., sepsis and septic shock), a pulmonary infection was the most common focus (60% vs. 59%), followed by an unknown infection focus (18% vs. 21%) and urogenital infection (14% vs. 7%). The distribution of the infectious focus did not statistically differ between both groups (*p* = 0.338). Accordingly, the distribution of blood-culture positive sepsis was comparable (45% vs. 49%; *p* = 0.386). Compared to patients admitted with sepsis without shock, the international normalized ratio (INR) (1.3 vs. 1.2; *p* = 0.001) and the aspartate aminotransferase (AST) levels (82 U/l vs. 43 U/l; *p* = 0.001) were significantly higher in patients presenting with a septic shock on admission.

**Table 2:** Sepsis-related data, follow-up data, and endpoints

	**All patients** (*n* = 349)		**Sepsis** (*n* = 197)		**Septic shock** (*n* = 152)		***p*-value**
**Sepsis scores,** median; (IQR)							
DIC	1	(0–2)	1	(0–2)	2	(1–3)	**0.001**
Acute physiology score	16	(12–21)	14	(9–19)	18	(14–23)	**0.001**
APACHE II	23	(18–29)	22	(15–27)	26	(21–31)	**0.001**
SOFA	11	(8–13)	10	(7–12)	13	(10–15)	**0.001**
ISARIC-4C-Mortality score	14	(12–16)	14	(12–16)	14	(12–16)	0.709
**Infection focus**, *n* (%)							
Pulmonary	207	(59.3)	118	(59.9)	89	(58.6)	0.338
Urogenital	38	(10.9)	27	(13.7)	11	(7.2)	
Intra-abdominal	32	(9.2)	15	(7.6)	17	(11.2)	
Wound	2	(0.6)	1	(0.5)	1	(0.7)	
Unknown	67	(19.2)	35	(17.8)	32	(21.1)	
SARS-CoV-2 infection, *n* (%)	40	(11.5)	31	(15.7)	9	(5.9)	**0.004**
**Microbiology**							
Positive blood cultures, *n* (%)	163	(46.7)	88	(44.7)	75	(49.3)	0.386
Number of positive blood cultures per patient, median; (IQR)	0.0	(0.0–2.0)	0.0	(0.0–2.0)	0.5	(0.0–2.0)	0.616
Gram-positive bacteria, *n* (%)							
Coagulase-negative Staphylococcus	65	(18.6)	37	(18.8)	28	(18.4)	0.932
Staphylococcus aureus	28	(8.0)	16	(8.1)	12	(7.9)	0.938
Enterococcus	24	(6.9)	14	(7.1)	10	(6.6)	0.847
Other	13	(3.7)	7	(3.6)	6	(3.9)	0.847
Gram-negative bacteria, *n* (%)							
Escherichia coli	28	(8.0)	13	(6.6)	15	(9.9)	0.265
Klebsiella pneumoniae	15	(4.3)	8	(4.1)	7	(4.6)	0.804
Pseudomonas aeruginosa	6	(1.7)	4	(2.0)	2	(1.3)	0.611
Other	13	(3.7)	8	(4.1)	5	(3.3)	0.706
**Antibiotic treatment at index, *n* (%)**							
Beta-lactam	290	(83.1)	165	(83.8)	125	(82.2)	0.707
Glycopeptide	17	(4.9)	10	(5.1)	7	(4.6)	0.839
Macrolide	18	(5.2)	11	(5.6)	7	(4.6)	0.682
Other	24	(6.9)	11	(5.6)	13	(8.6)	0.277
**Multiple organ support during ICU**							
Vasopressor support, *n* (%)	308	(88.3)	157	(79.7)	151	(99.3)	**0.001**
Dialysis during hospitalization, *n* (%)	152	(43.6)	66	(33.5)	86	(56.6)	**0.001**
Extracorporal membrane oxygenation, *n* (%)	24	(6.9)	14	(7.1)	10	(6.6)	0.847
**Respiratory status**							
Mechanical ventilation, *n* (%)	186	(53.3)	97	(49.2)	89	(58.6)	0.084
Invasive mechanical ventilation, *n* (%)	145	(41.5)	63	(32.0)	82	(53.9)	**0.001**
Duration of mechanical ventilation (days; mean, (range))	5	(1–16)	7	(1–17)	4	(1–14)	0.098
PaO_2_/FiO_2_ ratio (median; (IQR))	197	(135–292)	198	(138–297)	196	(132–291)	0.804
PaO_2_ (median; (IQR))	89	(74–120)	86	(71–116)	92	(78–126)	**0.038**
**Liver function**							
Acute liver failure, *n* (%)	30	(8.6)	10	(5.1)	20	(13.2)	**0.008**
**Renal function**, median; (IQR)							
Creatinine (mg/dl)	1.8	(1.1–3)	1.6	(1–2.9)	1.9	(1.4–3.1)	0.555
GFR (ml/min)	32.8	(19.1–57.9)	41.1	(19.9–65.3)	30.2	(18.5–46.9)	**0.002**
Urine output (ml)	800	(235–1575)	980	(413–1735)	530	(100–1313)	**0.022**
Dialysis (days)	0	(0–4)	0	(0–4)	2	(0–5)	0.254
**Baseline laboratory values**, median; (IQR)							
pH	7.37	(7.28–7.42)	7.39	(7.31–7.44)	7.33	(7.22–7.4)	0.389
Lactate (mmol/l)	2	(1.2–4.1)	1.3	(1–2)	3.6	(2.2–7.6)	**0.001**
Sodium (mmol/l)	139	(135–144)	139	(135–144)	139	(135–144)	0.396
Potassium (mmol/l)	4.2	(3.8–4.7)	4.2	(3.7–4.6)	4.2	(3.9–4.8)	**0.038**
Hemoglobin (g/dl)	9.9	(8.3–12.1)	9.9	(8.5–12.4)	10	(8.1–11.9)	0.473
WBC (10^6^/ml)	12.6	(8.1–18)	12.4	(8.3–17.1)	12.8	(7.7–19.7)	0.480
Platelets (10^6^/ml)	176	(106–268)	185	(124–265)	156	(85–269)	0.275
INR	1.2	(1.1–1.4)	1.2	(1.1–1.3)	1.3	(1.1–1.6)	**0.001**
Fibrinogen (g/l)	3.5	(2.5–5.6)	4.5	(2.9–6.3)	3.2	(2.1–4.7)	**0.016**
D-dimer (µg/l)	4.4	(1.6–16.1)	3.8	(1.4–10.4)	11.6	(4.2–32)	**0.001**
AST (U/l)	56	(29–127)	43	(25–79)	82	(43–187)	**0.011**
ALT (U/l)	31	(18–72)	27	(16–56)	38	(20–92)	0.061
Bilirubin (mg/dl)	0.9	(0.5–1.7)	0.8	(0.4–1.4)	1	(0.6–2)	0.111
Troponin I (µg/l)	0.2	(0–1)	0.1	(0–0.6)	0.5	(0.1–1.7)	0.304
NT-pro BNP (pg/ml)	2786	(897–7945)	2268	(775–6895)	4860	(1008–12,742)	0.149
Procalcitonin (ng/ml)	2.8	(0.7–18.4)	1.7	(0.6–11.8)	5.2	(1–31.2)	0.248
CRP (mg/l)	144	(83–220)	150	(97–225)	127	(79–219)	0.092
**Primary endpoint**							
All-cause mortality at 30 days, *n* (%)	180	(51.6)	81	(41.1)	99	(65.1)	**0.001**
Primary sepsis-related death at 30 days, *n* (%)	138	(76.7)	64	(79.0)	74	(74.7)	0.501
Primary non-sepsis-related death at 30 days, *n* (%)	42	(23.3)	17	(21.0)	25	(25.3)	
**Follow-up data**, *n* (%)							
ICU time (days; median; (IQR))	8	(3–20)	10	(4–21)	6	(3–17)	**0.042**
Death ICU, *n* (%)	168	(48.1)	70	(35.5)	98	(64.5)	**0.001**

*ALT* alanine aminotransferase, *APACHE II* Acute Physiology and Chronic Health Disease Classification System II, *AST* aspartate aminotransferase, *CRP* C-reactive protein, *DIC* disseminated intravascular coagulation, *GFR* glomerular filtration rate, *ICU* intensive care unit, *INR* international normalized ratio, *IQR* interquartile range, *NT-pro BNP* N-terminal pro-B-type natriuretic peptide, *SARS-CoV-2* severe acute respiratory syndrome coronavirus type 2, *SOFA* sepsis-related organ failure assessment score, *WBC* white blood cells

Level of significance *p* < 0.05. Bold type indicates statistical significance

### Association of CRP and PCT with clinical and laboratory data

Table 3 illustrates the correlation of the CRP and PCT on day 1 with clinical and laboratory data. CRP significantly correlated with creatinine (*r* = 0.148; *p* = 0.006) and procalcitonin (*r* = 0.331; *p* = 0.001). However, CRP did not correlate with other laboratory data, and no correlation of the CRP with sepsis-related scores was found. The PCT correlated with bilirubin (*r* = 0.198; *p* = 0.002) and creatinine (*r* = 0.255; *p* = 0.001). In line, PCT correlated with platelet count (*r* =  −0.172; *p* = 0.004) and activated partial thromboplastin time (aPTT) (*r* = 0.244; *p* = 0.001), as well as clinical parameters such as the PaO2/FiO2 ratio (*r* = 0.125; *p* = 0.048), mean arterial pressure (MAP) (*r* =  −0.170; *p* = 0.005), and days of mechanical ventilation (*r* =  −0.129; *p* = 0.034). In contrast, no correlation of the PCT with sepsis-related scores (i.e., APACHE II, acute physiology score, and SOFA score) was found (*p* > 0.005).

**Table 3:** Univariate correlations of CRP and PCT with laboratory and clinical parameters in all patients (*n* = 349) at day 1

	CRP		PCT	
	*r*	*p-*value	*r*	*p*-value
Age	0.016	0.766	0.050	0.409
BMI	0.031	0.575	−0.046	0.461
Creatinine (mg/dl)	0.148	**0.006**	0.255	**0.001**
Bilirubin (mg/dl)	0.049	0.401	0.198	**0.002**
Albumin (g/l)	−0.163	**0.004**	−0.123	0.058
CRP (mg/l)	-	-	0.331	**0.001**
Procalcitonin (ng/ml)	0.331	**0.001**	-	-
Hb (g/dl)	−0.066	0.216	−0.013	0.834
WBC (10^6^/ml)	0.016	0.770	0.100	0.101
Platelet count (10^6^/ml)	−0.025	0.644	−0.172	**0.004**
aPPT	0.088	0.128	0.244	**0.001**
PaO2/FiO2 ratio	0.015	0.785	0.125	**0.048**
MAP (mmHg)	−0.002	0.969	−0.170	**0.005**
Intensive care days	0.029	0.591	−0.118	0.052
Mechanical ventilation days	0.009	0.870	−0.129	**0.034**
Renal replacement days	0.070	0.192	0.033	0.591
Catecholamine use	−0.029	0.591	−0.013	0.831
SOFA score	0.002	0.970	0.105	0.083
Acute physiology score	−0.093	0.084	0.074	0.223
APACHE II score	−0.086	0.110	0.100	0.100

*APACHE II* acute physiology and chronic health evaluation II, *BMI* body mass index, *CRP* C-reactive protein, *INR* international normalized ratio, *aPPT* activated partial thromboplastin time, *SOFA* sepsis-related organ failure assessment score, *Hb* haemoglobin, *WBC* white blood cells

Level of significance *p* < 0.05. Bold type indicates statistical significance

### Diagnostic performance of CRP and PCT

**Fig. 1:**
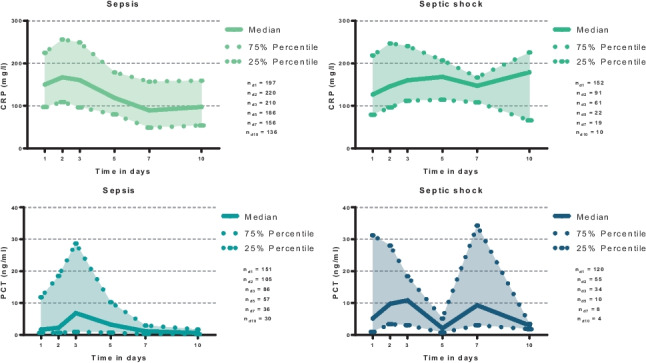
Distribution of CRP and PCT among patients with sepsis and septic shock during the first 10 days of sepsis onset (i.e., on days 1, 3, 5, 7, and 10). Data is presented as median with interquartile ranges

The distribution of the CRP and PCT levels patients admitted with sepsis or septic shock at days 1, 3, 5, 7, and 10 is presented in Fig. 1. In patients admitted with sepsis, the highest CRP level was seen on day 2 (median 167 (IQR 109–256) mg/l). Thereafter, a continuous decline of the CRP was observed until day 10. Initial CRP levels did not differ among patients with sepsis or septic shock (median CRP level day 1 in sepsis: 150 (97–225) mg/l; septic shock: 127 (79–219) mg/l; *p* = 0.092). However, in the presence of septic shock, CRP was shown to increase until day 10 of ICU hospitalization (median 179 (66–225) mg/l). In contrast, both in patients with sepsis and septic shock, the highest PCT levels were observed on day 3 (median PCT level day 3 in sepsis: 6.81 (0.95–28.68) ng/ml; septic shock: 10.83 (3–18.39 ng/ml; *p* = 0.820)).

C-statistics revealed comparable but poor diagnostic performance for PCT on day 1 (AUC = 0.590) and poor AUC for CRP (AUC = 0.440) to discriminate patients with septic shock from those with sepsis (*p* for AUC difference = 0.001). The AUCs for PCT were improved on day 7 and 10 (0.833–0.861) and moderate for CRP on day 5 to day 10 (AUC range 0.609–0.652) (Table 4). Furthermore, the diagnostic value of CRP (AUC 0.469–0.558) and PCT (AUC 0.535–0.654) with regard to blood-culture positive sepsis was poor during the first week of ICU hospitalization (Table 5).

**Table 4:** C-statistic for biomarkers to discriminate between sepsis and septic shock at days 1, 3, 5, 7, and 10

	CRP	PCT	*p*-value for AUC difference
Day 1	0.440 (0.379–0.501); *p* = 0.054	0.590 (0.520–0.659); *p* = 0.011	**0.001**
Day 1: Controls *n* = 197 patients with sepsis			
Day 3	0.507 (0.427–0.586); *p* = 0.875	0.568 (0.461–0.675); *p* = 0.248	0.401
Day 3: Controls *n* = 212 patients with sepsis			
Day 5	0.609 (0.486–0.733); *p* = 0.094	0.452 (0.278–0.616); *p* = 0.628	0.182
Day 5: Controls *n* = 188 patients with sepsis			
Day 7	0.628 (0.502–0753); *p* = 0.070	0.861 (0.695–1.000); ***p*** **= 0.002**	**0.038**
Day 7: Controls *n* = 158 patients with sepsis			
Day 10	0.652 (0.443–0.862); *p* = 0.109	0.833 (0.697–0.969); ***p*** **= 0.033**	0.265
Day 10: Controls *n* = 138 patients with sepsis			

Level of significance *p* < 0.05. Bold type indicates statistical significance

**Table 5:** C-statistic for biomarkers to discriminate between positive and negative blood cultures at days 1, 3, 5, 7, and 10

	CRP	PCT	*p*-value for AUC difference
Day 1	0.507 (0.446–0.568); *p* = 0.814	0.578 (0.510–0.647); ***p*** **= 0.026**	0.127
Day 3	0.558 (0.490–0.626); *p* = 0.098	0.616 (0.516–0.717); ***p*** **= 0.028**	0.349
Day 5	0.515 (0.436–0.593); *p* = 0.714	0.654 (0.520–0.787); ***p*** **= 0.033**	0.073
Day 7	0.510 (0.424–0.597); *p* = 0.812	0.535 (0.363–0.707); *p* = 0.700	0.802
Day 10	0.469 (0.374–0.563); *p* = 0.512	0.588 (0.385–0.791); *p* = 0.386	0.283

Level of significance p < 0.05. Bold type indicates statistical significance

### Prognostic performance of CRP and PCT

**Fig. 2:**
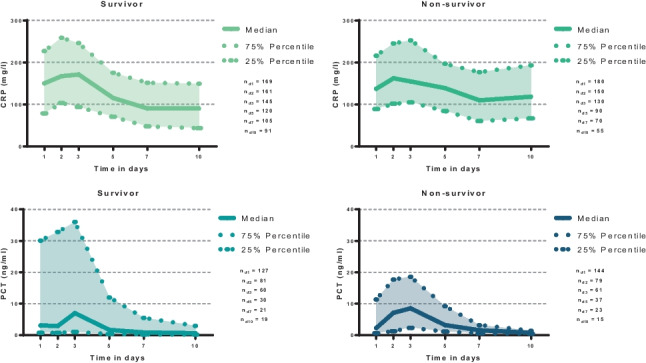
Distribution of CRP and PCT among 30-day survivors and non-survivors on days 1, 3, 5, 7, and 10. Data is presented as median with interquartile ranges

Overall risk of 30-day all-cause mortality was 52%. As illustrated in Fig. 2, the PCT levels did not significantly differ among 30-day survivors and non-survivors during the first 10 days of ICU hospitalization (median PCT day 1 in survivors: 3.08 (0.78–30.1) ng/ml; non-survivors: 2.28 (0.62–11.3) ng/ml; *p* = 0.083). Likewise, except for day 10 (median CRP day 10 in survivors: 90 (43–149) mg/l; non-survivors: 118 (67–193); *p* = 0.041), the CRP levels also did not significantly differ among both groups during the first 10 days of ICU hospitalization (median CRP day 1 in survivors: 150 (78–227) mg/l; non-survivors: 137 (88–216) mg/l; *p* = 0.269).

The prognostic AUCs of CRP (range of AUC 0.475 to 0.596) and PCT (range of AUC 0.456 to 0.550) were poor at all time points to predict all-cause mortality at 30 days (Table 6).

**Table 6:** C-statistic for biomarkers at days 1, 3, 5, 7, and 10 to discriminate between non-survivors and survivors of the 30-day time interval

	CRP	PCT	*p*-value for AUC difference
Day 1	0.475 (0.414–0.536); *p* = 0.416	0.456 (0.386–0.525); *p* = 0.208	0.685
Day 1: Controls *n* = 197 patients with sepsis			
Day 3	0.507 (0.439–0.575); *p* = 0.840	0.512 (0.407–0.617); *p* = 0.820	0.937
Day 3: Controls *n* = 212 patients with sepsis			
Day 5	0.558 (0.479–0.636); *p* = 0.153	0.550 (0.401–0.698); *p* = 0.488	0.922
Day 5: Controls *n* = 188 patients with sepsis			
Day 7	0.568 (0.481–0.655); *p* = 0.129	0.531 (0.352–0.710); *p* = 0.724	0.707
Day 7: Controls *n* = 158 patients with sepsis			
Day 10	0.596 (0.501–0.692); *p* = 0.052	0.521 (0.313–0.730); *p* = 0.835	0.505
Day 10: Controls *n* = 138 patients with sepsis			

Level of significance *p* < 0.05. Bold type indicates statistical significance

**Fig. 3:**
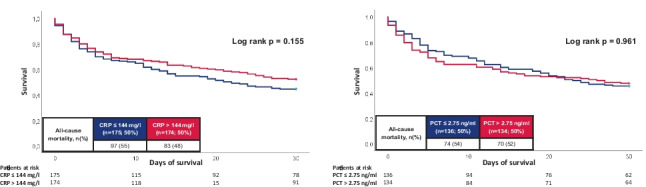
Kaplan–Meier curves for CRP and PCT with regard to all-cause mortality at 30 days within the entire study cohort

When stratified for the median CRP and PCT levels on day 1, all-cause mortality occurred in 55% of the patients with CRP ≤ 144 mg/l and 48% with CRP > 144 mg/l at 30 days. Accordingly, risk of all-cause mortality was not affected by CRP (log rank *p* = 0.155; HR = 0.999; 95% CI 0.998–1.001; *p* = 0.203) (Fig. 3, left panel). Accordingly, 30-day all-cause mortality was not affected by initial PCT level (52% vs. 54%; log rank *p* = 0.961; HR = 0.998; 95% CI 0.993–1.003; *p* = 0.500) (Fig. 3, right panel).

### Prognostic impact of CRP and PCT during course of sepsis and septic shock

**Fig. 4:**
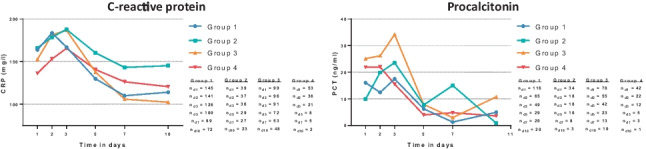
Dynamic changes of CRP (left panel) and PCT (right panel) within pre-specified subgroups: (group 1) patients with initial sepsis without progression to septic shock; (group 2) patients with initial sepsis with progression to septic shock; (group 3) patients with initial septic shock with clinical improvement to sepsis without shock; (group 4) patients with initial septic shock without clinical improvement to sepsis without shock

When stratified for the dynamic change of the clinical improvement/impairment, CRP was shown to decrease in patients with sepsis without shock (group 1) (mean CRP day 1 164 ± 96 mg/l; mean CRP day 10 114 ± 82 mg/l; *p* = 0.001), patients with initial sepsis with progression to septic shock (group 2) (mean CRP day 1 166 ± 112 mg/l; mean CRP day 10 145 ± 81 mg/l; *p* = 0.437) and to decrease in patients with septic shock improving to sepsis without shock (group 3) (mean CRP day 1 153 ± 108 mg/l; mean CRP day 10 102 ± 70 mg/l; *p* = 0.001), as well as in patients with septic shock without clinical improvement (group 4) (mean CRP day 1 136 ± 85 mg/l; mean CRP day 10 121 ± 60 mg/l; *p* = 0.797) (Fig. 4). In line, PCT levels were shown to decrease during the first 10 days of ICU hospitalization within the analyzed subgroups.

**Fig. 5:**
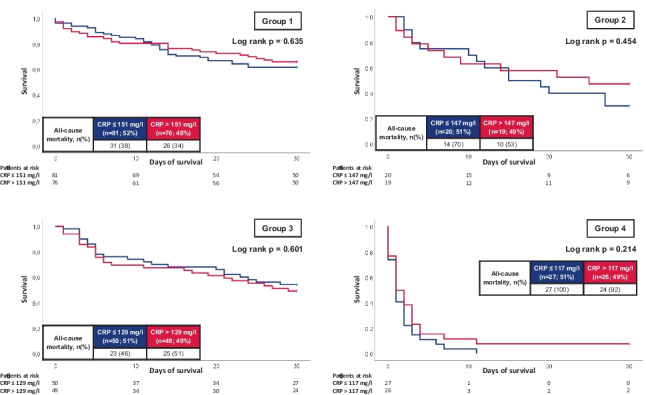
Kaplan–Meier curves for CRP with regard to all-cause mortality at 30 days stratified by dynamic disease progression/improvement during ICU hospitalization

**Fig. 6:**
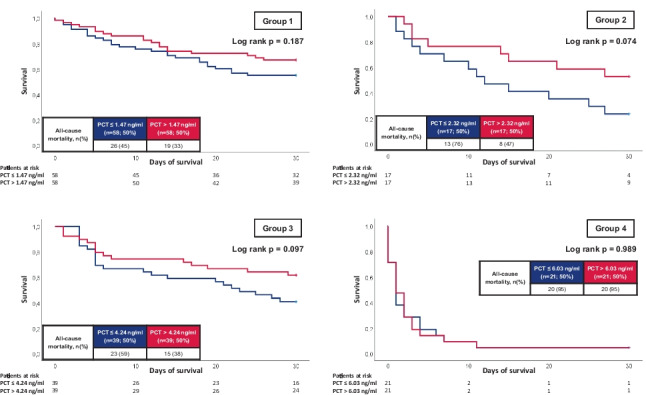
Kaplan–Meier curves for PCT with regard to all-cause mortality at 30 days stratified by dynamic disease progression/improvement during ICU hospitalization

When stratified for clinical improvement or impairment according to the above-mentioned subgroups, both CRP and PCT were not shown to be associated with 30-day all-cause mortality within pre-specified subgroups (i.e., initial sepsis without impairment; initial sepsis progressing to septic shock; initial septic shock improving to sepsis without shock; and initial septic shock without clinical improvement) (Figs. 5 and 6).

### Diagnostic and prognostic value of CRP and PCT after exclusion of patients with COVID-19 disease

Even after exclusion of patients with sepsis related to COVID-19 disease, the diagnostic value of CRP on day 1 to discriminate patients with septic shock (AUC = 0.436; 95% CI 0.371–0.500; *p* = 0.052) was poor. Accordingly, the diagnostic value of PCT was poor to moderate (AUC = 0.578; 95% CI 0.505–0.651; *p* = 0.036). Furthermore, both the prognostic value with regard to 30-day all-cause mortality of CRP (AUC = 0.467; 95% CI 0.311—0.402; *p* = 0.311) and PCT (AUC = 0.455; 95% CI 0.382–0.528; *p* = 0.221) was poor and comparable to the AUCs including COVID-19 patients (*p* > 0.05) (data not shown).

## Discussion

The present study comprehensively investigates the diagnostic and prognostic role of CRP and PCT in patients admitted with sepsis or septic shock. This data suggests CRP has a poor diagnostic accuracy to discriminate between patients with sepsis and septic shock compared to PCT. PCT was shown to have a good diagnostic accuracy on day 7 and 10 (AUC 0.833–0.861). In contrast, CRP and PCT were shown to have a poor prognostic value with regard to 30-day all-cause mortality. Both CRP and PCT levels did not affect the risk of 30-day all-cause mortality in patients with sepsis and septic shock. Even when stratified for clinical improvement or impairment during course of ICU hospitalization, CRP and PCT were not associated with the risk of 30-day all-cause mortality.

The diagnostic value of CRP and PCT was investigated within various registries with conflicting findings. Silvestre et al. investigated the diagnostic and prognostic role of CRP in 158 patients with sepsis, severe sepsis, and septic shock using a prospective registry. They found no association of the CRP concentration on day 1 with the severity of the sepsis. Furthermore, higher CRP concentrations were not associated with an increased risk of ICU mortality [13]. In line, the diagnostic and prognostic values of CRP, PCT, and presepsin were investigated by Lee et al. including 420 patients with non-infectious organ failure, sepsis, and septic shock. Presepsin and PCT were able to discriminate the diagnosis septic shock from sepsis, whereas CRP was not shown to discriminate septic shock from sepsis. Furthermore, presepsin was shown to be predictive for 28-day all-cause mortality, which was still proven after multivariable Cox regression analyses. In contrast, CRP and PCT levels did not differ among 28-day survivors and non-survivors; however, their analyses were based on a single biomarker assessment [5]. The present study confirms no prognostic impact of CRP and PCT levels in patients with sepsis and septic shock - which is line with reports from our study group from the year 2004 [3]. But still in the sepsis-3 era modern intensive care for patients with septic shock, these commonly applied inflammatory markers do not reveal a relevant diagnostic and prognostic impact in this setting. 

Of note, a microbacterial-proven sepsis is only reported in 30–40% of patients admitted with sepsis or septic shock [6]. Both CPR and PCT were not shown to be predictive for microbacterial-proven sepsis in 157 patients with suspected sepsis (AUC 0.53 and 0.55). Furthermore, the decline of 5 days to baseline CRP and PCT did not significantly differ among 28-day non-survivors compared to survivors [17]. The present study specifically focused on the diagnostic and prognostic impact of CRP and PCT during the course of ICU treatment. Of note, specifically PCT was shown to have good diagnostic accuracy from day 7 to day 10 (AUC 0.833–0.861), whereas the diagnostic value during the early stages of sepsis and septic shock was poor. In line, the present study suggested poor diagnostic value of both CRP and PCT with regard to blood-culture positive sepsis.

Various studies investigated the prognostic role of CRP and PCT in patients with sepsis with conflicting findings. Koozi et al. found an increased risk of mortality in patients with CRP > 100 mg/l including 851 patients with sepsis [18]. In line, the prognostic value of CRP was studied in 313 patients admitted to the ICU, which was not restricted to patients with sepsis. Increasing CRP levels were associated with more severe organ dysfunction, longer ICU stay, and increased risk of all-cause mortality. Furthermore, an increase of CRP after 48 h was associated with an increased risk of all-cause mortality [19]. These findings are in line with a study by Wang et al. that included 576 ICU patients, of which 6% presented with sepsis. The authors demonstrated a reliable prognostic value of the CRP to predict ICU mortality (AUC = 0.65) [20].

Although PCT is often postulated to have a higher prognostic value than CRP, PCT was not shown to be associated with 28-day all-cause mortality in 371 patients with signs of infection [21]. In line, a study by Gornet et al. found both PCT and CRP to have moderate discrimination for bacteraemia in 459 patients with suspected infection (AUC 0.68 and 0.65), whereas the discrimination for 28-day all-cause mortality was poor and even inferior to systolic blood pressure and pulse oximetry [22]. The prognostic value of CRP, PCT, and the neutrophil-to-lymphocyte ratio (NLR) was comprehensively investigated within a retrospective study including 146 patients with bloodstream infection and sepsis according to the sepsis-3 criteria. Using multivariable Cox regression analyses, the authors demonstrated that especially PCT and NLR were associated with 28-day mortality. Both PCT and NLR revealed a reliable prognostic discrimination (AUC 0.830 and 0.791) for 28-day all-cause mortality [23]. However, no risk stratification was performed according to the severity of sepsis and no dynamic changes of CRP, PCT, and NLR during the course of sepsis were assessed. The findings of the present study are in line with previous studies that found a poor discrimination for early sepsis-related mortality based on a single biomarker, specifically in the absence of assessment of dynamic changes of biomarker levels [24].

The kinetics of CRP and PCT levels during the course of septic shock were investigated within a study by Bahloul et al. including 60 patients, demonstrating a fall in CRP or PCT level was associated with improved prognosis [25]. On the other hand, CRP and PCT were inferior to interleukin-6 (IL-6) predicting treatment success in 328 patients with sepsis when re-assessed after 48–72 h [26]. Within the present study, a continuous decline in CRP from day 1 to day 10 was observed, which was irrespective of clinical improvement or impairment. CRP levels were also demonstrated to decrease in patients with initial sepsis progressing to septic shock during the course of ICU treatment. These patients were shown to be at increased risk of death (corresponding mortality rate 62% versus 36% in patients without clinical impairment). This data suggests a decline of CRP levels may not an appropriate tool to detect clinical improvement during course of ICU treatment. This may further emphasize the need to investigate the prognostic role of blood-derived biomarkers that may correlate with clinical improvement/impairment during course of sepsis or septic shock.

In contrast, the NLR is linked to elevated levels of proinflammatory cytokines in patients with sepsis or septic shock and was shown to predict outcomes in patients with infectious disease, cancer, and in patients following surgery. As a result of its rapid increase (commonly within 6 h), the NLR may be useful to predict sepsis severity [27]. Li et al. recently demonstrated the prognostic accuracy for short-term death in 302 septic patients was improved, when combining established sepsis-scores (such as the SOFA and APACHE II scores) with the NLR [28]. However, on the contrary, the NLR may be increased in various clinical conditions and further studies did not observe an interaction of the NLR and risk prediction in patients with sepsis or septic shock [29, 30]. Related to the rather small number of studies investigating the prognostic role of the NLR in septic patients compared to CRP and PCT, the NLR was infrequently assessed within the present real-life registry and no follow-up measurements of the NLR were performed. Therefore, the present study underlines the need to further assess inflammatory biomarkers in patients with sepsis and septic shock.

In conclusion, the present study suggests a superior predictive value of the PCT compared to the CRP for the diagnosis of a septic shock. In contrast, both CRP and PCT had a poor predictive value for 30-day all-cause mortality. CRP and PCT were shown to decrease irrespective of clinical improvement or impairment, but did not affect 30-day all-cause mortality when stratified for clinical improvement or impairment during course of ICU hospitalization.

## Study limitations

This study has several limitations. Due to the single-center and observational study design, results may be influenced by measured an unmeasured confounding. Although it was shown that IL-6 is associated with prognosis in critically ill and septic patients, IL-6 is not measured in clinic routine at our institution and was therefore beyond the scope of this registry [31]. With regard to the diagnostic value of inflammatory markers, no control group with healthy individuals was considered. Furthermore, side effects regarding treatment with antibiotics were not assessed for the present study. Finally, the effect of inflammatory markers on long-term outcomes was beyond the scope of the present study.

## Data Availability

The datasets used and/or analyzed during the current study are available from the corresponding author on reasonable request.
